# Supplementary material for: Centrifugal multimaterial 3D printing of multifunctional heterogeneous objects
Source: Nat Commun. 2022 Dec 24;13:7931. doi: 10.1038/s41467-022-35622-6 (PMC9789974; doi:10.1038/s41467-022-35622-6)
Supplement: Supplementary file 1 — Supplementary Information [file 41467_2022_35622_MOESM1_ESM.pdf]

Supplementary Information

Centrifugal Multimaterial 3D Printing of Multifunctional Heterogeneous Objects

Jianxiang Cheng[1,2], Rong Wang[1,2], Zechu Sun[1,2], Qingjiang Liu[1,2], Xiangnan He[1,2], Honggeng Li[1,2], Haitao Ye[1,2,3], Xingxin Yang[4], Xinfeng Wei[1,2], Zhenqing Li[1,2], Bingcong Jian[1,2], Weiwei Deng[1,5], Qi Ge[1,2*]

[1]Shenzhen Key Laboratory of Soft Mechanics & Smart Manufacturing, Southern University of Science and Technology, Shenzhen, 518055, China

[2]Department of Mechanical and Energy Engineering, Southern University of Science and Technology, Shenzhen 518055, China

[3]Department of Mechanical Engineering, City University of Hong Kong, Kowloon, Hong Kong SAR, China

[4]School of Electronics and Communication Engineering, Guangzhou University, Guangzhou 510006, China

[5]Department of Mechanics and Aerospace Engineering, Southern University of Science and Technology, Shenzhen 518055, China

*Corresponding Author: Qi Ge, e-mail: geq@sustech.edu.cn.

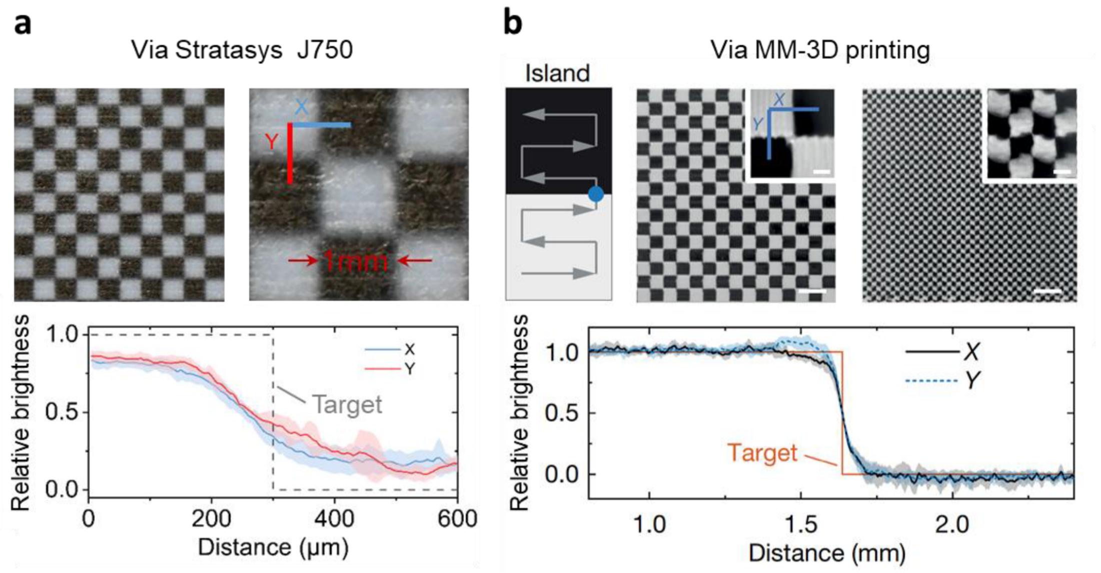

Supplementary Fig. 1 | Comparison on the two-material transition zoom. **a**, A checkboard pattern printed by an inkjet-based commercial multimaterial 3D printer (Stratasys J750). The two-material transition zoom is about 200 μm, but the transition is not very sharp. **b**, A checkboard pattern printed by multimaterial multinozzle 3D printing system reported by Lewis et al.[9] The two-material transition zoom is about 200 μm. The distortions on the white and black squares can be seen. Shaded regions represent the standard deviation.

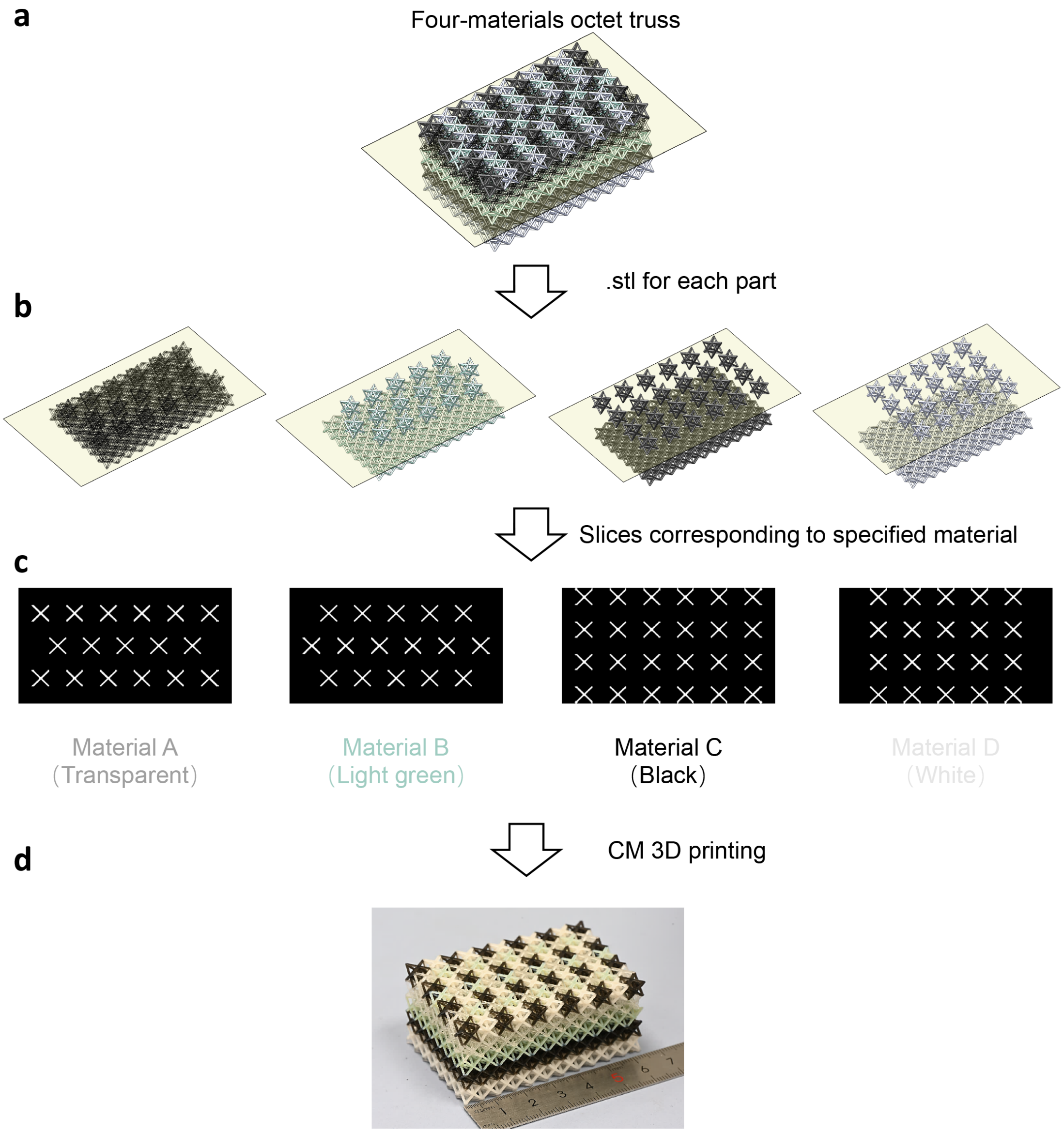

Supplementary Fig. 2 | Slicing strategy for printing an octet truss structure consisting of four materials. a, A structure consisting of four parts was created in Solidworks, and the .stl file for each part was generated by saving the structure as assembly. **b,** The .stl files were then uploaded to a self-developed MATLAB code which created slices for each part and numbered all the slices. **c,** Example of the created slices for different materials at the same layer. **d,** The four-material structure was printed by sending the slices for different materials in sequence to the CM 3D printing system. The .stl files were then uploaded to a self-developed MATLAB code which created slices for each part and numbered all the slices.

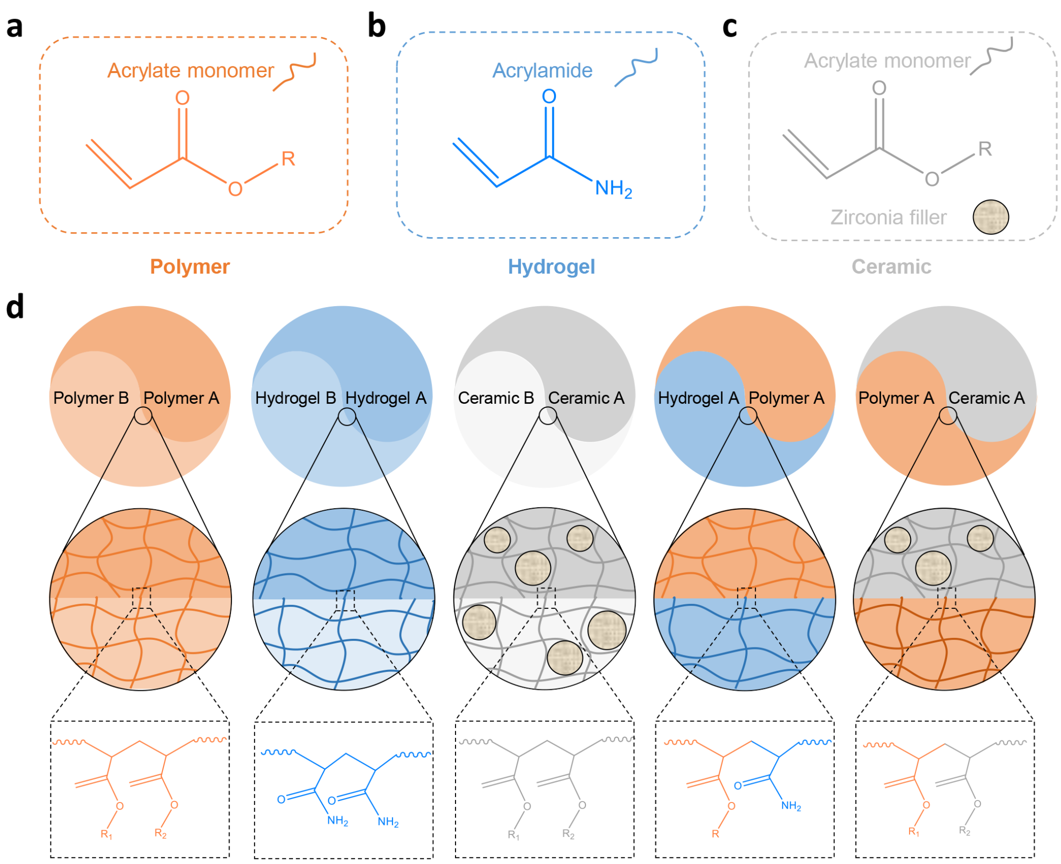

Supplementary Fig. 3 | Multifunctional heterogeneous objects bonding mechanism. **a-c**, Possible chemical structure for polymer, hydrogel, and ceramic polymer resin. **d**, Possible chemical bonding for the polymer-polymer, hydrogel-hydrogel, ceramic-ceramic, hydrogel-polymer, or polymer-ceramic interfaces.

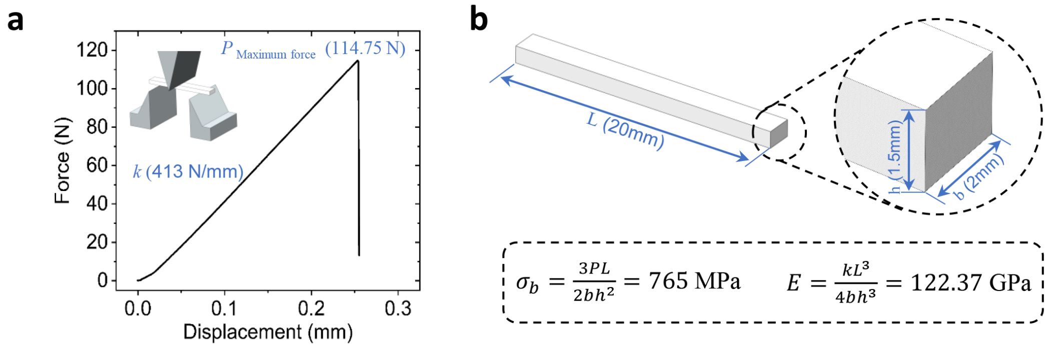

Supplementary Fig. 4 | Three-point bending test to measure the mechanical property of printed ZrO_2 ceramic. a, Measured force-displacement relation. **b**, Detailed process on calculating the flexible strength σ_b and Young's modulus E of the ceramic sample.

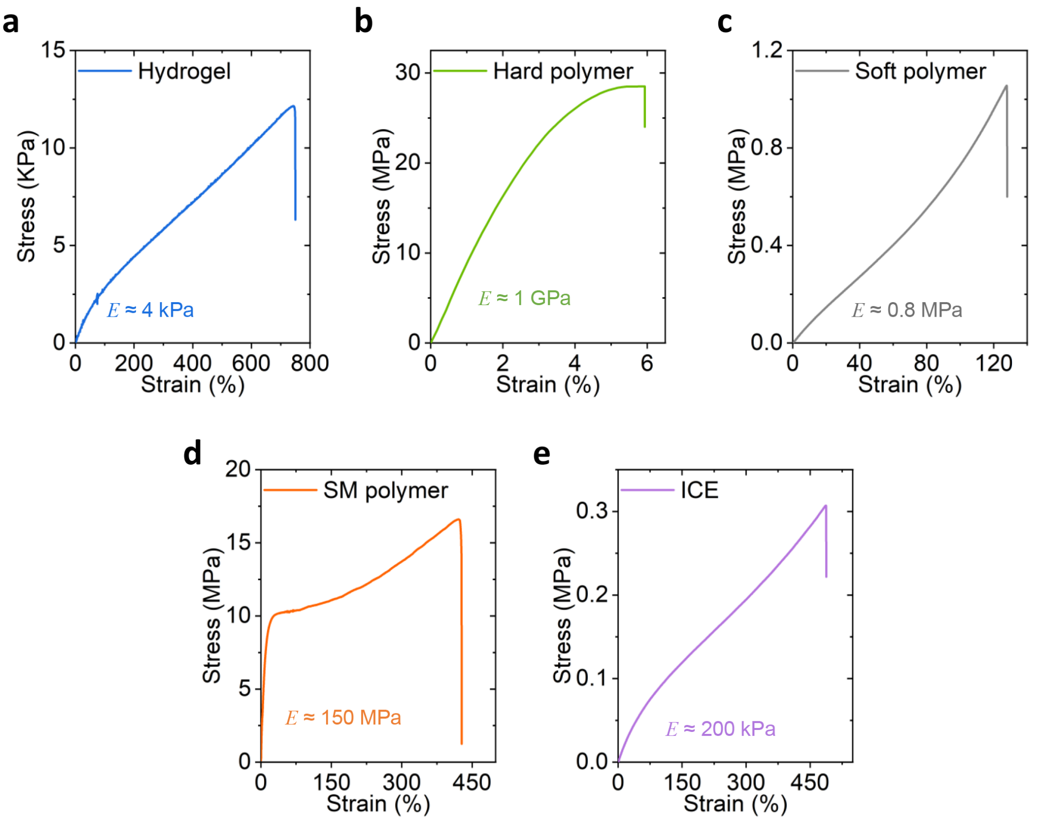

Supplementary Fig. 5 | Room temperature uniaxial tensile tests for the materials used in Fig. 1c-f. a, Hydrogel. **b**, Hard polymer. **c**, Soft polymer. **d**, Shape memory (SM) polymer. **e**, Ionic conductive elastomer (ICE).

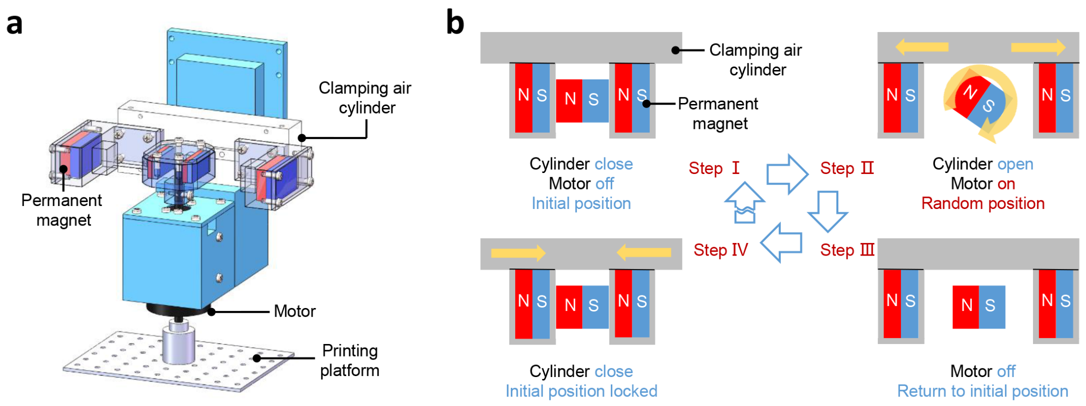

Supplementary Fig. 6 | Illustration and working mechanism of the rotating printing platform. a, Detailed design of the rotating printing platform. **b,** Work mechanism to describe how to make the printing platform to precisely return to its initial position after spinning.

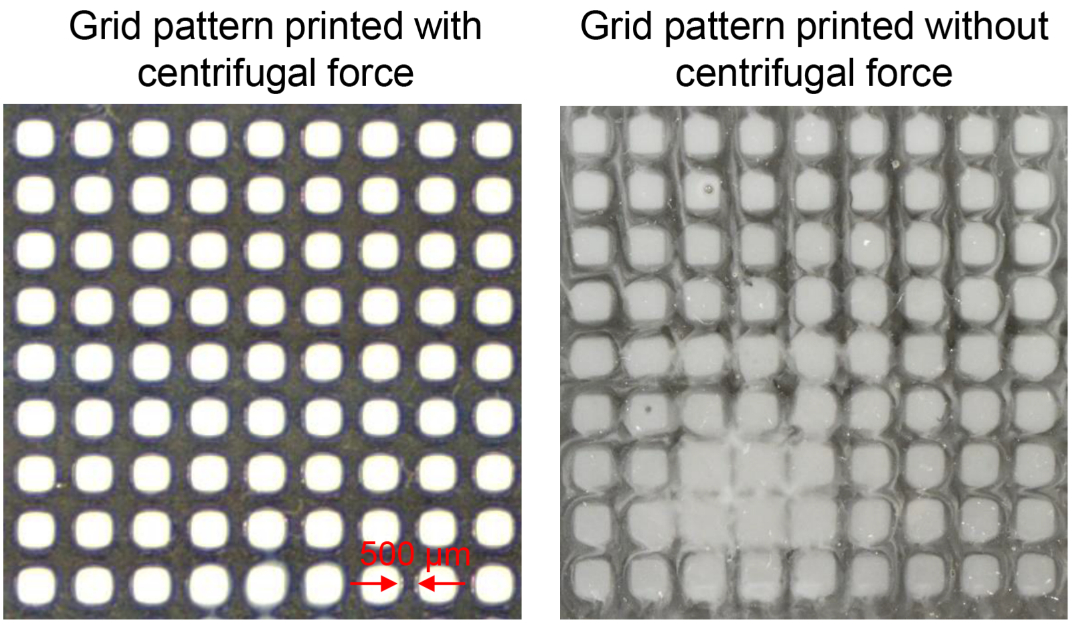

Supplementary Fig. 7 | Comparison on printing a grid pattern with/without centrifugal force.

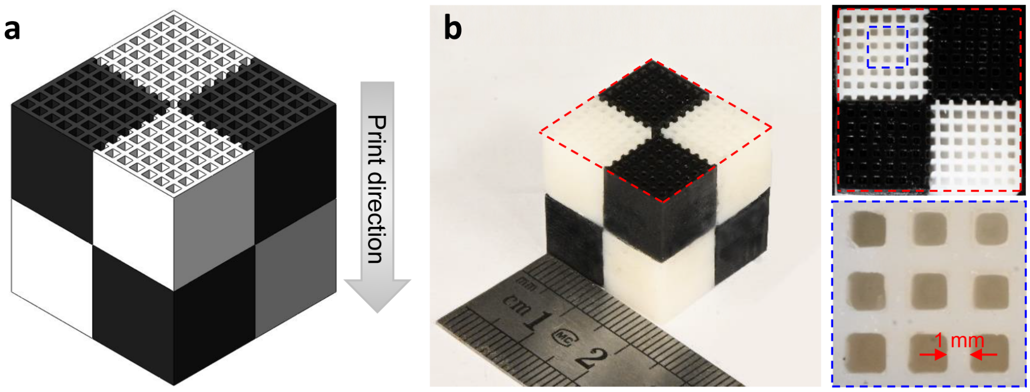

Supplementary Fig. 8 | Printing a multimaterial structure with channels perpendicular to the centrifugal force directions. a, CAD model of the multimaterial structure. **b,** Snapshot of the structure printed via the CM 3D printing system.

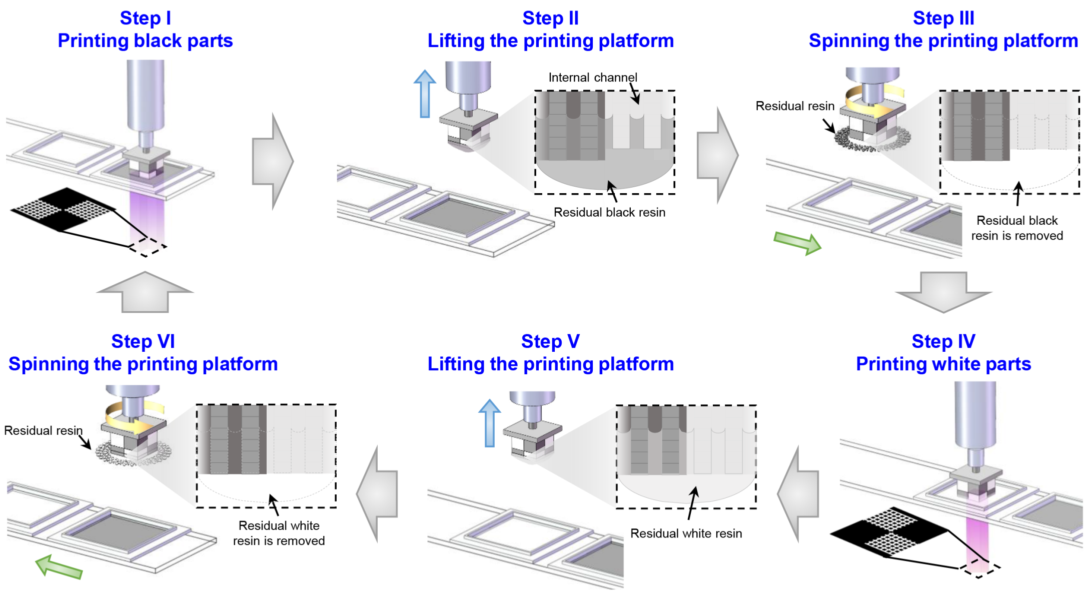

Supplementary Fig. 9 | Detailed steps to print a multimaterial structure which has two-material parts at each layer and internal channels perpendicular to the centrifugal force directions.

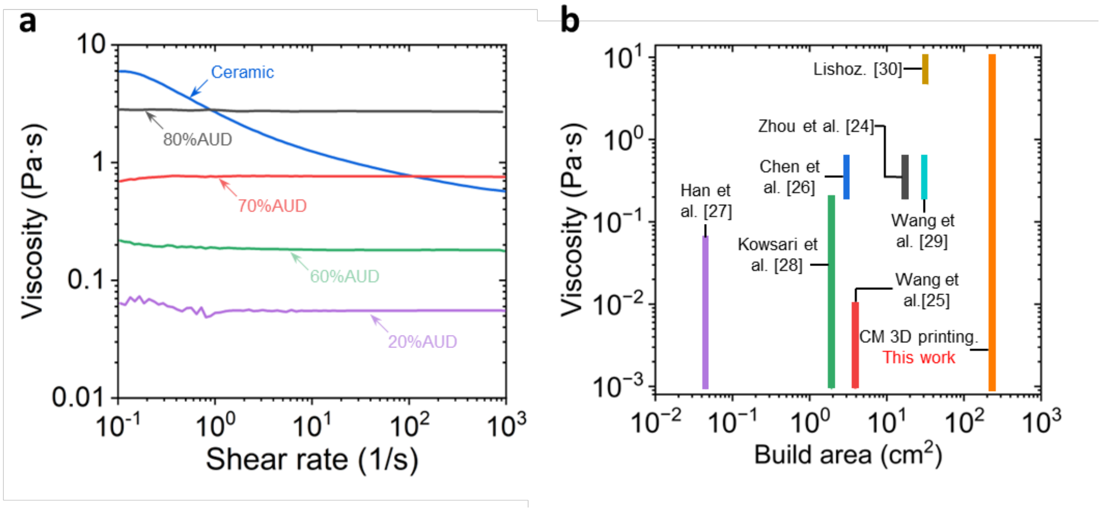

Supplementary Fig. 10 | Viscosity of polymer resins. a, Viscosity of the polymer resins used to investigate the effect of spinning speed and time on the thickness of the residual resin. Ceramic resin was prepared by mixing 1,6-hexanediol diacrylate, PEGDA and ZrO_2 ceramic powders. Newtonian polymer resins were prepared by *t*BA and AUD with different content of AUD. Details on the polymer resin preparation can be found in Materials. **b,** Comparison on the relation between the viscosity range of polymer resin and build area of different DLP-based multimaterial 3D printing methods.

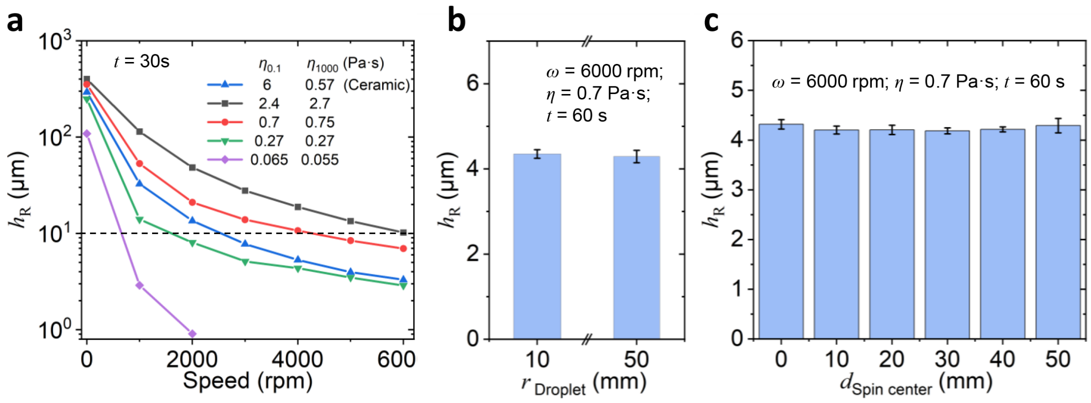

Supplementary Fig. 11 | Investigation on the thickness of residual resin. **a,** Thickness of residual resin (h_R) varies with spinning speed for the resins with different viscosity. h_R was measured after 30 s spinning. **b,** Experiments show that the radius of polymer resin droplet ($r_{Droplet}$) does not affect the h_R. **c,** Experiments show that h_R is independent of the location where it is measured ($d_{spin\ center}$: distance to the spin center).

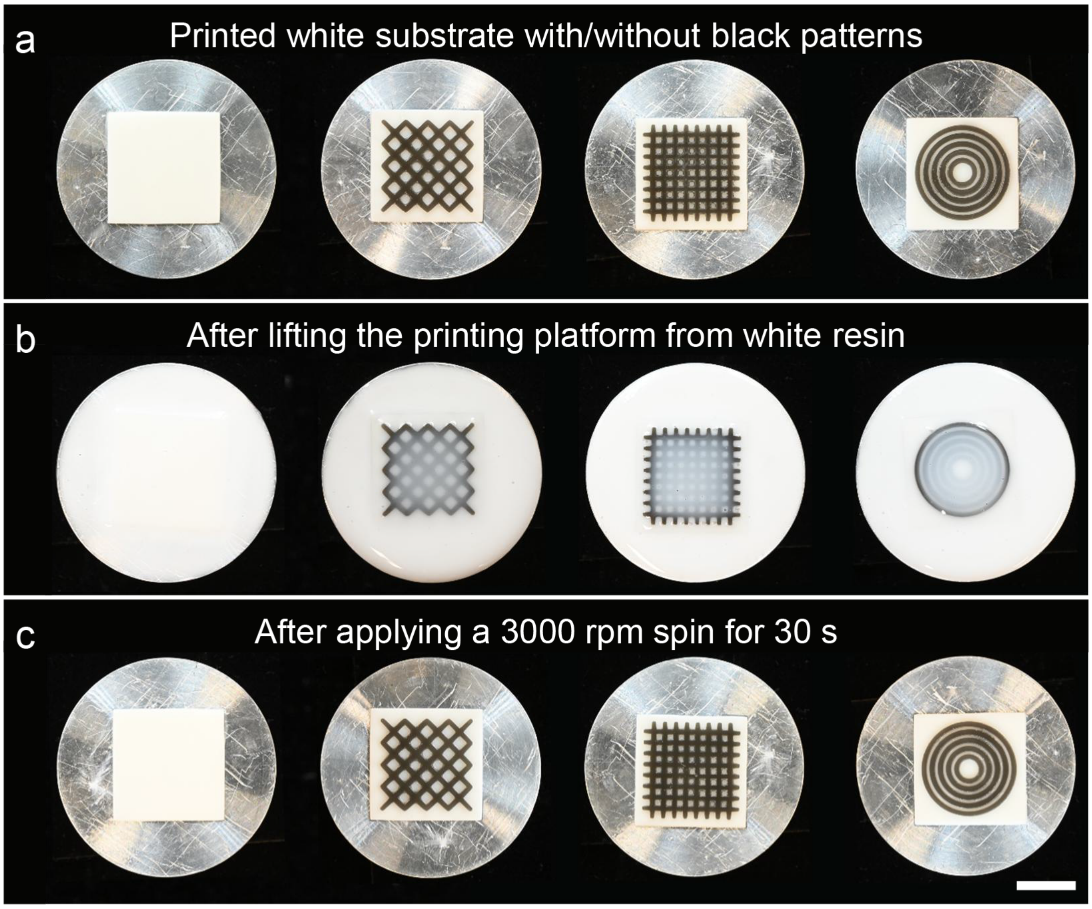

Supplementary Fig. 12 | Effect of printed patterns on the efficiency of removing residual resin via centrifugal force. a, Snapshots of a printed white substrate, and printed white substrates with different black patterns. **b,** Snapshots of the printed structures which were just left from a white resin (viscosity: 0.2 Pa·s). **c,** Snapshots of the printed structures where the white resins were removed by applying 3000 rpm spin for 30 s. Video of the experiment can be found in Supplementary Video 7. Scale bars in **c,** 10 mm.

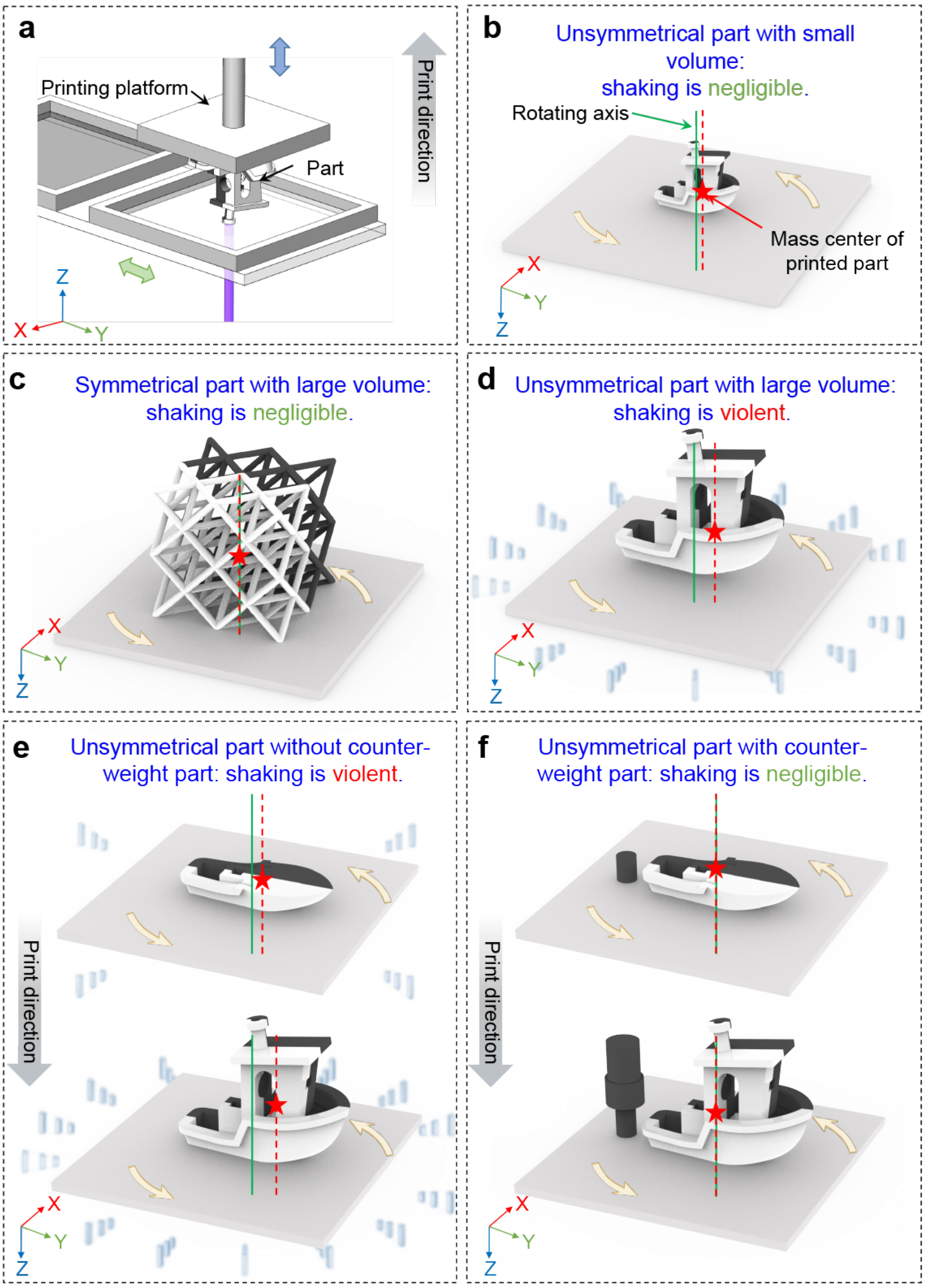

Supplementary Fig. 13 | Shaking during the process of printing unsymmetrical parts. a, CM 3D printing system. **b,** Shaking is negligible when printing a unsymmetrical part with small volume. **c,** Shaking is negligible when printing a symmetric part with large volume. **d,** Shaking becomes violent when printing a unsymmetrical part with large volume. **e,** Violent shaking worsens during printing as the mass center of the printed part drifts away from the rotating axis. **f,** Violent shaking can be eliminated by printing extra counter-weight part which makes the mass center of total printed parts on the rotating axis.

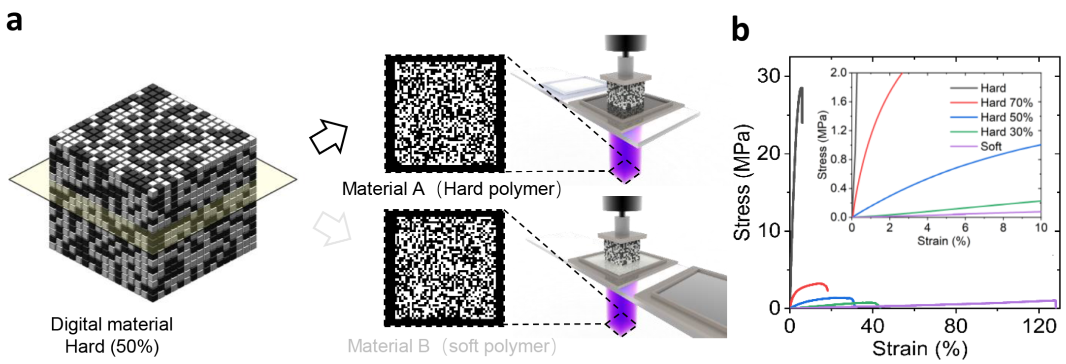

Supplementary Fig. 14 | Digital materials printed by the CM 3D printing system. a, Illustration on the design of a digital material consisting of 50% hard polymer. The hard and soft voxels are randomly distributed in each layer. **b,** Uniaxial tensile testing results of the digital materials with different hard material contents.

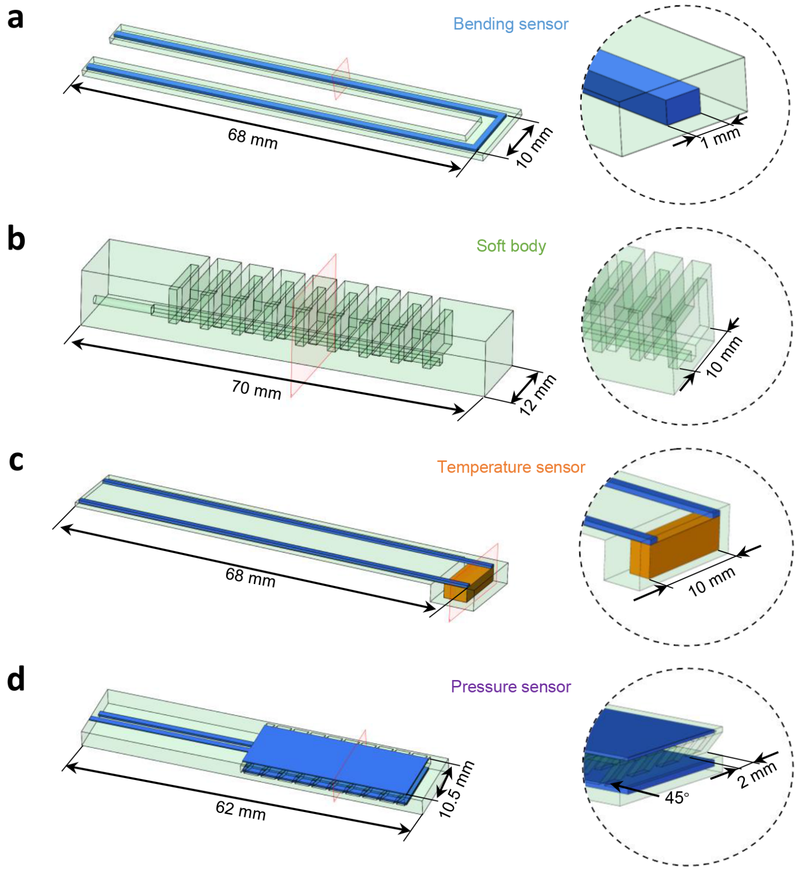

Supplementary Fig. 15 | Detailed geometric parameters of each unit on the SPA. a, Bending sensor. **b,** Soft body. **c,** Temperature sensor. **d,** Pressure sensor.

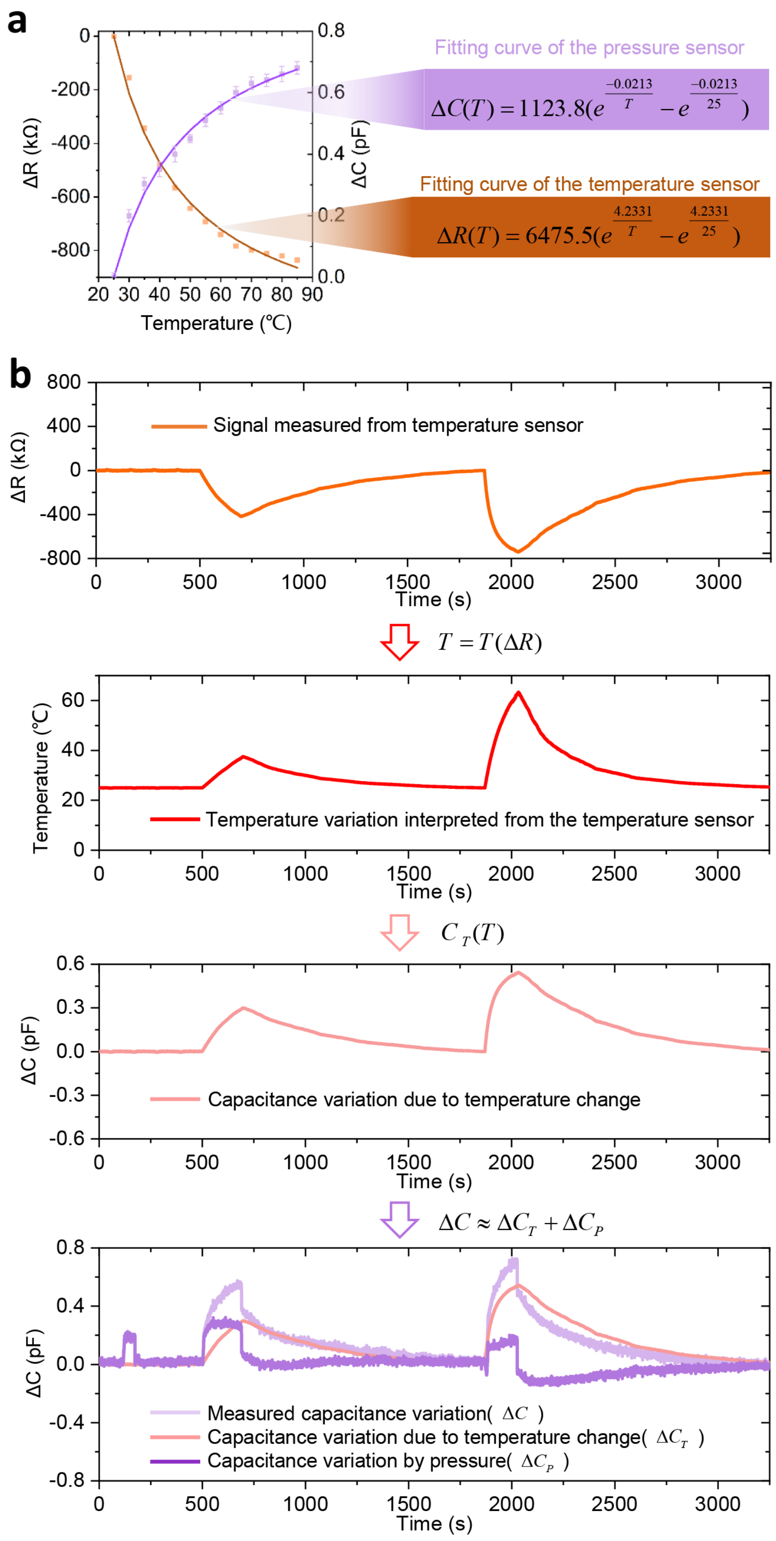

Supplementary Fig. 16 | Approach to decouple the temperature effect on the capacitive pressure sensor. a, Temperature effect on both the temperature and pressure sensor. **b,** Detailed decoupling approach.

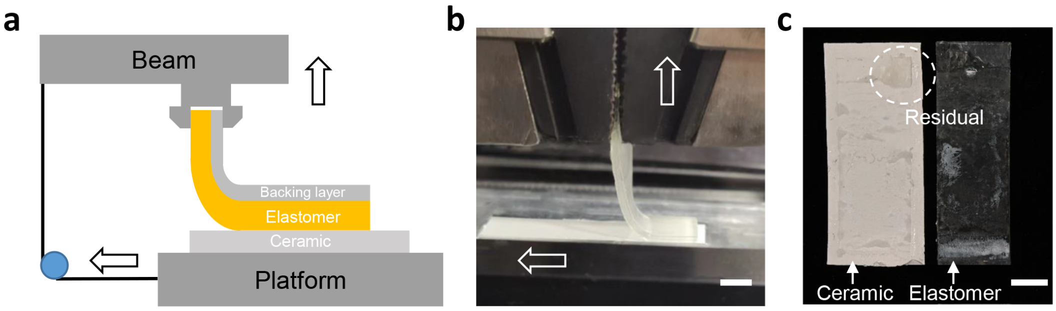

Supplementary Fig. 17 | 90° peeling test to measure the interfacial toughness between elastomer and ceramic green body. a, Schematic illustration of a 90° peeling test. **b,** Snapshot showing the process of a 90° peeling test. Scale bars in **b,** 5 mm. **c,** Image of a fractured sample implying that the fracture is cohesive. Scale bars in **c,** 5 mm.

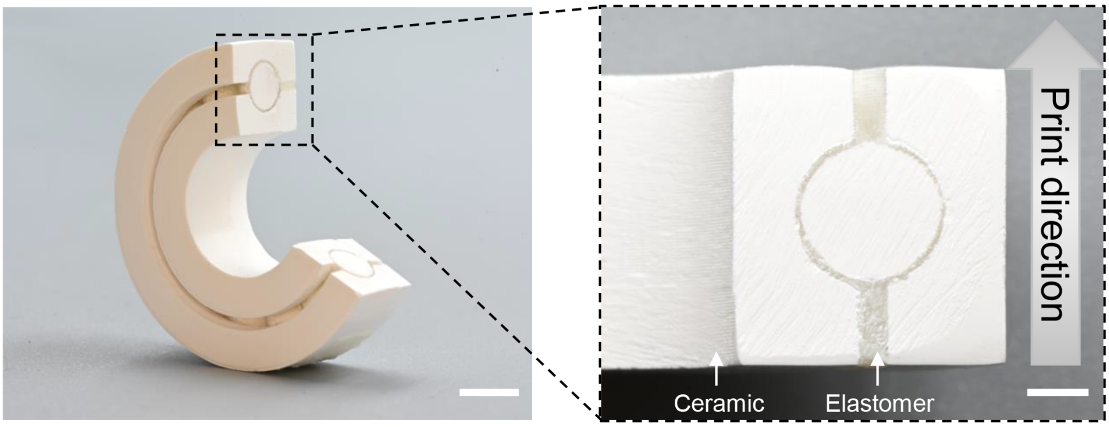

Supplementary Fig. 18 | A printed two-third of ceramic bearing clearly showing that the rollers are supported by elastomer. Scale bars: left, 5 mm; right, 2 mm.

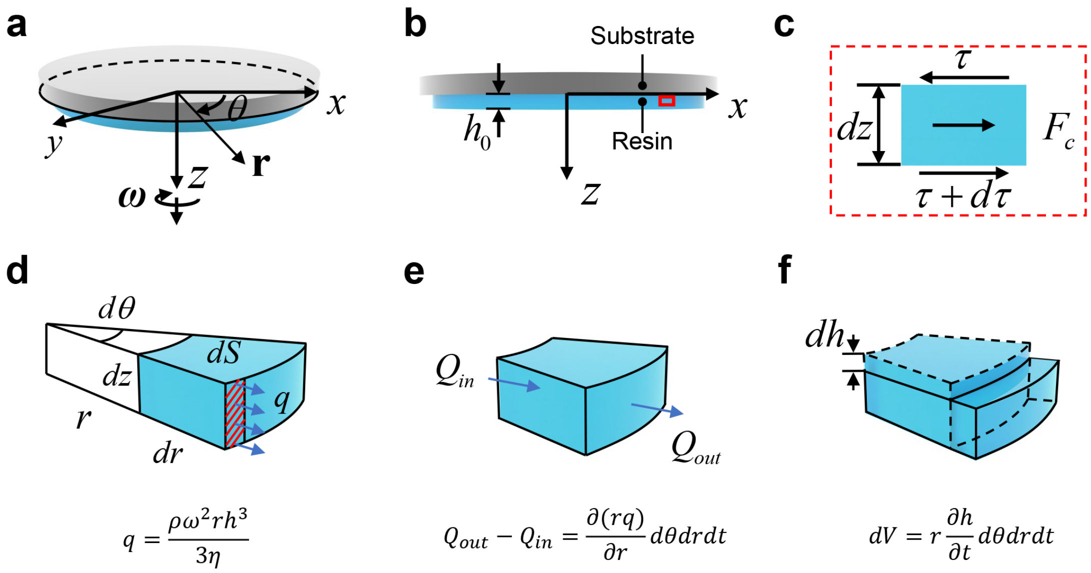

Supplementary Fig. 19 | Schematic illustrations on modeling of the process of removing polymer resin through spinning the printing platform. a, Coordinates for modeling. **b,** Initial condition. **c,** Force diagram on an infinitesimal element. **d,** Radial flow q per unit length of circumference. **e,** Net outflow ΔQ ($\Delta Q = Q_{out} - Q_{in}$) of the liquid through the infinitesimal element during the time increment dt. **f,** Volume change ΔV due to the net outflow.

Name	Build area (cm^2)	Optical resolution (μm)	Viscosity (Pa.s)
Zhou et al.[24]	17.28 (4.8 cm×3.6 cm)	47	0.2~0.6
Wang et al.[25]	3.9 (2.6 cm×1.5 cm)	60	0.2~0.6
Chen et al.[26]	3 (2 cm×1.5 cm)	20	0.2~0.6
Han et al.[27]	0.045 (0.3 cm×0.15 cm)	5	0.001~0.06
Kowsari et al.[28]	1.92 (1.6 cm×1.2 cm)	15	0.001~0.2
Wang et al.[29]	29.93 (7.3 cm×4.1 cm)	38	0.001~0.01
Lithoz.[30]	32.68 (7.6 cm×4.3 cm)	40	5~10
This work	234 (18 cm×13 cm)	25	0.001~10

Supplementary Table 1 | Comparison on build area, optical resolution and viscosity range between previously reported DLP-based multimaterial 3D printer and CM 3D printer in this work.

Printing methods	3D Printer	Resolution	Maximum Printing Area	Printing Mode	Speed of printing two materials in a one layer
DLP	Zhou. et al.[24]	Optical resolution: 47 µm	48 mm × 36 mm	Direct Projection	2.88 mm^2/s
	Wang et al.[25]	Optical resolution: 60 µm	26 mm ×15 mm	Direct Projection	0.65 mm^2/s
	Chen et al.[26]	Optical resolution: 20 µm	20 mm ×15 mm	Direct Projection	Cannot be estimated
	Han et al.[27]	Optical resolution: 5 µm	3 mm ×1.5 mm	Direct Projection	0.18 mm^2/s
	Kowsari et al.[28]	Optical resolution: 15 µm	16 mm × 12 mm	Direct Projection	12.8 mm^2/s
	Wang et al.[29]	Optical resolution: 38 µm	73 mm × 41 mm	Direct Projection	Cannot be estimated
	Lithoz et al.[30]	Optical resolution: 40 µm	76 mm × 43 mm	Direct Projection	Cannot be estimated
	CM 3D Printer in this work	Optical resolution: 25 µm	48 mm × 27 mm	Direct Projection	10.8 mm^2/s
			180 m ×130 mm	Direct Projection + Scanning	39 mm^2/s
		Optical resolution: 75 µm	150 m × 160 mm	Two-light-engine Projection	200 mm^2/s
DIW	MM 3D Printer[9]	Printing Nozzle Diameter: 200 µm	725 mm × 650 mm	1-Nozzle Printing	2.9 mm^2/s
				8-Nozzle Printing	18.8 mm^2/s
Polyjet	Stratasys J750[8]	Build Resolution: +/- 100 µm	490 mm × 390 mm	-	315 mm^2/s

Supplementary Table 2 | Comparison on the speed of printing two materials in one layer between other multimaterial 3D printers and CM 3D printer in this work.

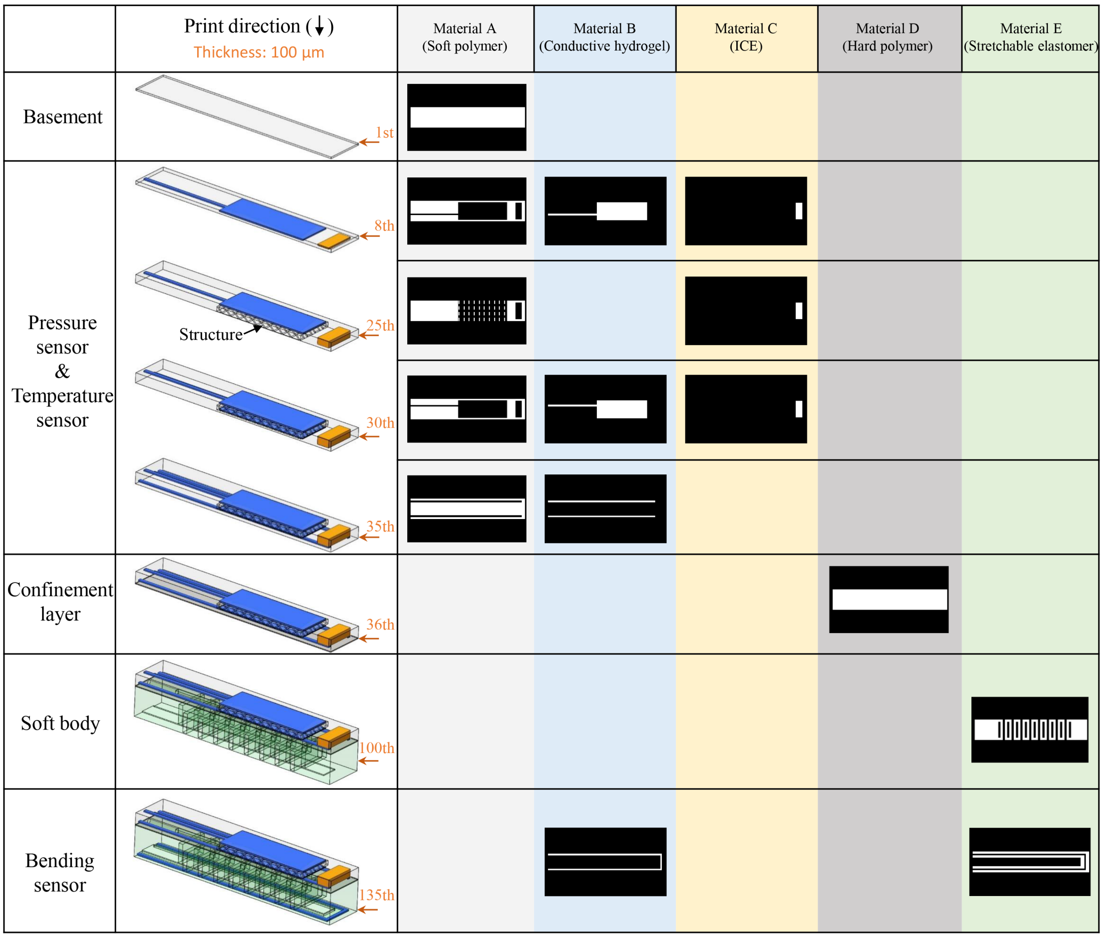

Supplementary Table 3 | Five different materials used to construct different layers of the SPA during the CM 3D printing process.

Structure	Material	Layer Thickness	Curing time	Spinning speed	Spinning time
Fig. 1a, Fig. 2b,c, d, f and Fig. 3a,b,c (polymer with polymer)	Vero black	200 µm	7 s	6000 rpm	30 s
	Vero white	200 µm	12 s	6000 rpm	30 s
Fig. 1b (polymer with polymer)	Vero black	100 µm	5 s	6000 rpm	30 s
	Vero white	100 µm	3 s	6000 rpm	30 s
	Vero clear	100 µm	3 s	6000 rpm	30 s
	ABS plus	100 µm	3 s	6000 rpm	30 s
Fig. 1c (Hydrogel with polymer)	Hydrogel	100 µm	6 s	3000 rpm	30 s
	Red hydrogel	100 µm	6 s	3000 rpm	30 s
Fig. 1d (polymer with polymer)	Vero white	100 µm	5 s	6000 rpm	30 s
	Vero clear	100 µm	3 s	6000 rpm	30 s
Fig. 1e (polymer with polymer)	SMP	100 µm	8 s	6000 rpm	30 s
	Vero Clear	100 µm	3 s	6000 rpm	30 s
Fig. 1f (polymer with polymer)	ICE	100 µm	5 s	6000 rpm	30 s
	Agilius	100 µm	3 s	6000 rpm	30 s
Fig. 1g (ceramic with ceramic)	Ceramic	50 µm	10 s	6000 rpm	30 s
	Blue ceramic	50 µm	10 s	6000 rpm	30s
Fig. 3a, b, c (polymer with polymer)	Vero black	100 µm	5 s	6000 rpm	30 s
	Vero white	100 µm	3 s	6000 rpm	30 s
Fig. 3d, f (polymer with polymer)	Agilius	100 µm	3 s	6000 rpm	30 s
	Vero black	100 µm	5 s	6000 rpm	30 s
Fig. 3h, I, j (polymer with polymer)	Hydrogel	100 µm	6 s	3000 rpm	30 s
	Agilius	100 µm	3 s	6000 rpm	30 s
	Vero black	100 µm	5 s	6000 rpm	30 s
Fig. 4c, d, f, h, j (polymer with hydrogel)	Hydrogel	100 µm	6 s	3000 rpm	30s
	Elastomer	100 µm	5 s	6000 rpm	30 s
	Vero clear	100 µm	3 s	6000 rpm	30 s
	Agilius	100 µm	3 s	6000 rpm	30 s
	ICE	100 µm	5 s	6000 rpm	30 s
Fig. 5a, b, d, f, g, h, i (polymer with ceramic)	Agilius	100 µm	3 s	6000 rpm	30 s
	Ceramic	50 µm	10 s	6000 rpm	30 s

Supplementary Table 4 | Printing parameters for printing all the structures in this work.
