# Supplementary material for: Centrifugal multimaterial 3D printing of multifunctional heterogeneous objects
Source: Nat Commun. 2022 Dec 24;13:7931. doi: 10.1038/s41467-022-35622-6 (PMC9789974; doi:10.1038/s41467-022-35622-6)
Supplement: Supplementary file 3 — Description of Additional Supplementary Files [file 41467_2022_35622_MOESM3_ESM.pdf]

Description of Additional Supplementary Files

File Name: Supplementary Movie 1
Description: a CM 3D Printed two material Kelvin foam structure where a soft polymer layer is sandwiched by two hard polymer layers

File Name: Supplementary Movie 2
Description: a CM 3D printed Miura-origami sheet where the hard polymer panels are connected by the shape memory (SM) polymer hinges which allows the flat Miura-origami sheet to be programmed to a 3D shape.

File Name: Supplementary Movie 3
Description: a CM 3D printed flexible ionic conductive (IC) octet truss consisting of an IC elastomer (ICE) core surrounded by the nonconductive soft polymer part

File Name: Supplementary Movie 4
Description: printing and multimaterial switching processes of the CM 3D printing system.

File Name: Supplementary Movie 5
Description: comparison on the printing platform with or without spinning.

File Name: Supplementary Movie 6
Description: an experimental demonstration shows that a 1000 rpm spinning of the printing platform does not damage an extremely soft hydrogel structure (Young modulus: 4 kPa), but a 6000 rpm spinning of the printing platform does damage the extremely soft hydrogel structure.

File Name: Supplementary Movie 7
Description: an experimental demonstration shows that under the same spinning condition (speed: 3000 rpm, time: 30 s), the residual resins on all the printed patterns can be quickly removed.

File Name: Supplementary Movie 8
Description: a CM 3D printed SPA with multiple sensing capabilities. The bending and pressure sensors response differently when the soft robotic gripper grabs nothing, a duck, and an orange, while the resistance of the temperature sensor is constant

File Name: Supplementary Movie 9
Description: a CM 3D printing ceramic ball bearing could rotate freely without resistance after sintering process.

File Name: Supplementary Movie 10

Description: demonstration of a turbine where the CM 3D printed ceramic bearing connects the metal shaft and impeller. The impeller can spin at a high speed due to the low friction of the ceramic bearing
